# Evaluation of fully automated ApoE4 proteotyping for *APOE* ε4 genotype estimation in the FINDERI cohort

**DOI:** 10.1002/dad2.70362

**Published:** 2026-05-18

**Authors:** Hans‐Wolfgang Klafki, Carlotta Derad, Maike Hohberg, Thomas Asendorf, Esselmann Hermann, Mohammed M. Hassan, Niels Hansen, Christopher M. Celano, Stephanie Heinemann, Ingo Kutschka, Hassina Baraki, Monika Sadlonova, Christine AF von Arnim, Jens Wiltfang, Oliver Wirths

**Affiliations:** ^1^ Department of Psychiatry and Psychotherapy University Medical Center Göttingen, Georg‐August‐University Göttingen Germany; ^2^ Department of Medical Statistics University Medical Center Göttingen, Georg‐August‐University Göttingen Germany; ^3^ Department of Psychiatry Massachusetts General Hospital Boston USA; ^4^ Department of Psychiatry Harvard Medical School Boston USA; ^5^ Department of Geriatrics University Medical Center Göttingen, Georg‐August‐University Göttingen Germany; ^6^ University Heart Center Basel, University Hospital Basel Basel Switzerland; ^7^ Department of Psychosomatic Medicine and Psychotherapy University Medical Center Göttingen, Georg‐August‐University Göttingen Germany; ^8^ Department of Cardiovascular and Thoracic Surgery University Medical Center Göttingen, Georg‐August‐University Göttingen Germany; ^9^ German Center for Cardiovascular Research (DZHK) Göttingen Germany; ^10^ German Center for Neurodegenerative Diseases (DZNE) Göttingen Germany; ^11^ Department of Medical Sciences, Neurosciences and Signaling Group Institute of Biomedicine (iBiMED), University of Aveiro Aveiro Portugal

**Keywords:** Alzheimer's disease, apolipoprotein E, ARIA risk, dementia, genotype, plasma, proteotype, risk factor

## Abstract

**INTRODUCTION:**

Carriers of the ε4 allele of the apolipoprotein E (*APOE*) gene have an increased risk for Alzheimer's disease (AD) and amyloid‐related imaging abnormalities (ARIAs) upon anti‐amyloid beta (Αβ) immunotherapy. Measuring apoE4 and pan‐apoE proteins in blood plasma for apoE4 proteotyping may offer an alternative to *APOE* genotyping.

**METHODS:**

We assessed apoE4 proteotyping accuracy in 479 participants of the prospective FINd DElirium RIsk factors (FINDERI) study in patients undergoing cardiac surgery and compared results to quantitative polymerase chain reaction (qPCR) genotyping.

**RESULTS:**

Proteotype–genotype discordance occurred in 8 of 479 participants (1.67%). Five of 17 proteotype homozygotes were genotypically heterozygous. Replacing manufacturer provided cut points with custom data‐driven thresholds substantially improved classification performance.

**DISCUSSION:**

We confirm the reported overall high classification performance of apoE4 proteotyping but underscore the need to re‐evaluate the generalizability of the cut points provided with the assay kits. Misclassification of heterozygous *APOE* ε4 carriers as homozygous could erroneously exclude eligible patients from anti‐amyloid therapies.

## Highlights

- Plasma apoE4 proteotyping was compared to *APOE* ε4 genotyping results in participants of the FINDERI study cohort.
- In 8 cases, the proteotypic classification was discordant with genotyping.
- Five out of 17 subjects proteotyped as homozygous ε4 carriers were genotyped as heterozygous
- Replacing manufacturer‐provided cutoffs for the proteotyping by data‐driven thresholds improved the classification accuracy.
- The observations raise concerns regarding the generalizability of the cutoffs provided with the assay kits.

## 1 BACKGROUND

The ε4 allele of the apolipoprotein E gene (*APOE* ε4) is the most important genetic risk factor for late‐onset Alzheimer's disease (AD).^1^, ^2^, ^3^ Furthermore, patients with AD who have one or two copies of *APOE* ε4 and are treated with the monoclonal anti‐amyloid beta (Aβ) antibodies lecanemab (Leqembi) or donanemab (Kisunla) have an increased risk of developing amyloid‐related imaging abnormalities with edema or effusions (ARIA‐E) or amyloid‐related imaging abnormalities with hemosiderin deposits (ARIA‐H).^4^, ^5^

In the European Union, Leqembi and Kisunla are currently authorized for the treatment of patients with mild cognitive impairment (MCI) and early dementia due to AD who have none or only a single copy of *APOE* ε4.^6^, ^7^ Assessment of the *APOE* genotype is thus required for evaluating eligibility for anti‐Aβ immunotherapy in Europe.

Immunoassays for quantification of apoE4 and pan‐apoE protein levels in blood plasma to determine the “apoE proteotype” may offer potential alternatives to classical *APOE* genotyping. In two recent studies, fully automated Lumipulse apoE4 and pan‐apoE chemiluminiscent enzyme immunoassays were evaluated against *APOE* ε4 genotyping in a clinical sample of patients with cognitive symptoms from Sweden (*n* = 164)^8^ and in patients from Italy (*n* = 65), who underwent *APOE* genotyping for suspected AD.^9^ In both studies, the apoE4 proteotype determinations showed high performance in classifying *APOE* ε4 genotype. However, although Musso et al. observed no overlap in the plasma apoE4/pan‐apoE ratio between *APOE* ε4 non‐carriers, heterozygous and homozygous subjects, and “perfect concordance with genetic testing,”^9^ Dittrich et al. found a small number of outliers in their sample resulting in overlaps among the groups and misclassification of three individuals.^8^ In both studies, it was stated that the respective sample sizes were limited and replication studies were required for verification of the findings.

Here we report on the assessment of the Lumipulse apoE4 and pan‐apoE blood plasma immunoassays against polymerase chain reaction (PCR) *APOE* genotyping in plasma samples from the FINd DElirium RIsk factors (FINDERI) study, which aims to identify risk factors for delirium and cognitive decline in cardiac surgery patients.^10^ Our study sample comprising 479 heart surgery patients distinguishes this investigation from previous work (see above)^8^, ^9^ and may expand available information regarding the generalizability of published findings into a new setting.

**RESEARCH IN CONTEXT**

1. **Systematic review**: The authors reviewed the literature using PubMed, meeting abstracts and other online sources. The recent approval of anti‐amyloid therapies for Alzheimer's disease in Europe requires knowledge of the *APOE* genotype, as homozygosity of the ε4 allele is an exclusion criterion. Recently, determination of the ApoE proteotype has been suggested as a possible alternative to classical *APOE* genotyping.
2. **Interpretation**: We compared ApoE4 proteotyping using Lumipulse plasma immunoassays with the results of qPCR *APOE* genotyping in a cohort of 479 patients aged > 50 years who underwent cardiac surgery. Our results demonstrate discordant results in a subset of patients. The classification performance of the proteotyping for *APOE ε4* genotype estimation was improved when the manufacturer‐provided cutoffs were replaced by data‐driven thresholds. Our findings raise concerns regarding the generalizability of the manufacturer‐provided cutoffs
3. **Future directions**: Future research should investigate the performance of ApoE4 proteotyping for *APOE* ε4 genotype estimation in other and larger samples to further evaluate the utility and generalizability of cutoffs across different populations and sites.

## 2 METHODS

### 2.1 Study participants and sample collection

We recruited *n* = 504 patients with various cardiac conditions in the prospective, observational, interdisciplinary, and monocentric FINDERI study,^10^ all of whom underwent elective cardiac surgery between February 2021 and October 2022, in the Department of Cardiovascular and Thoracic Surgery at the University Medical Center Göttingen, Germany. The study's inclusion criteria were age ≥50 years, admission to the hospital for cardiac surgery, and ability to speak and understand German and thus give informed consent. Participants with significant cognitive impairment, such as dementia or other problems that could impair verbal communication, were excluded from the study. All participants provided written informed consent. The study concurs with the Declaration of Helsinki, and an ethics vote was obtained when the first patient was recruited (AZ20/11/20, amendment 21/07/21). Furthermore, the study was registered as a clinical trial with the German Clinical Trials Register (DRKS00025095). A separate ethics vote (AZ9/2/2016) was obtained for the processing, storage, and preservation of blood biomaterial samples for this study. Blood samples were collected 1 to 2 days before cardiac surgery from *n* = 479 patients and processed according to a standardized protocol. Blood sample processing included ethylenediaminetetraacetic acid (EDTA) plasma centrifugation at 2000 × *g* for 10 min with aliquoting into 500 µL portions and storage at −80°C until further use. The samples were then stored in our biobank at the Department of Psychiatry and Psychotherapy, University Medical Center Göttingen. The clinical variables were age, gender, an index for comorbidity (Charlson Comorbidity Index),^11^ body mass index (BMI), any renal insufficiency (based on medical history), the need for acute dialysis, cognitive status as measured by the Montreal Cognitive Assessment (MoCA),^12^ and the presence of MCI defined by a MoCA score of fewer than 26 of 30 points.

### 2.2 *APOE* genotyping

The genetic *APOE* status was determined using a quantitative real‐time PCR protocol as described previously.^13^ In brief, total DNA was extracted from 100 µL non‐nucleated blood with the DNeasy Blood & Tissue kit (Qiagen). The following primers were used: ApoE_112C 5′‐CGGACATGGAGGACGGTGT‐3′; ApoE_112R 5′‐CGGACATGGAGGACGGTGC‐3′; ApoE_158C 5′‐CTGGTACACCTGCCAGGCA‐3′; ApoE_158R 5′‐CTGGTACACCTGCCAGGCG‐3′. *APOE* primers were combined in three different reaction mixtures to allow amplification of APOE2 (ApoE_112C/ApoE_158C), APOE3 (ApoE_112C/ApoE158R) and APOE4 (ApoE_112R/ApoE_158R) variants, and all samples were measured for all three primer combinations.^13^ Analyses were carried out using the iTaq Universal SYBR Green Supermix (BIO‐RAD) in a total volume of 20 µL on a CFX Connect Real‐Time PCR system (BIO‐RAD). The PCR cycling conditions were as follows: initial denaturation at 95°C for 10 min, followed by 40 cycles of 95°C for 15 s and 65°C for 40 s. Negative as well as positive controls from samples with known *APOE* genotype were included in all assay runs.

### 2.3 ApoE4 proteotyping

For determination of the apoE4 proteotype, apoE4‐ and pan‐apoE proteins in EDTA blood plasma samples were quantified in single measurements on a fully automated Lumipulse 600 G (Fujirebio) chemiluminescence analyzer using the research‐use‐only (RUO) Lumipulse G ApoE4 and Lumipulse G pan‐ApoE assays (product numbers 81453 and 81449, Fujirebio). Following the assay kit instructions, the apoE4/pan‐apoE ratio (%) was calculated, and study participants were classified into non‐carriers (apoE4/pan‐apoE ratio <5%, heterozygous (5% ≤ ratio < 75%), or homozygous (ratio ≥75%).

### 2.4 Statistical analysis

The research questions addressed in this exploratory study were not part of the pre‐specified goals of the FINDERI study.

Plasma concentrations of apoE4 and pan‐apoE, as well as the apoE4/pan‐apoE ratio, were stratified by *APOE* genotype, and group differences were assessed using Kruskal–Wallis tests followed by Dunn's post hoc multiple comparisons.

Participants were proteotypically classified as non‐carriers, heterozygotes, or homozygotes using the assay kit cutoffs (apoE4/pan‐apoE ratio <5%, 5% to <75%, and ≥75%, respectively). Concordance between proteotype classification and qPCR‐determined *APOE* genotype was assessed at the individual level. Diagnostic performance of the apoE4/pan‐apoE ratio for binary classification of *APOE* ε4 positivity (presence of ≥1 ε4 allele) was evaluated using the manufacturer‐recommended cutoff, with sensitivity, specificity, positive and negative predictive values (PPV and NPV), and diagnostic accuracy reported with 95% confidence intervals (CIs). Performance of the 75% cutoff for discrimination of homozygous versus heterozygous *APOE* ε4 carriers was evaluated in the carrier subgroup (*n*  =  135).

When the proteotypic apoE4/pan apoE ≥75% cutoff is used to identify genotypically *APOE* ε4 homozygous individuals, it can be interpreted as targeting one of two distinct decision objectives: discrimination of homozygous carriers versus all other individuals in an unselected population (a screening scenario with substantial class imbalance), or finer allele dose discrimination between heterozygous and homozygous carriers. As these represent fundamentally different classification problems, both were evaluated separately.

To contextualize the performance of the manufacturer recommended upper cutoff across these scenarios, receiver‐operating characteristic (ROC) analyses were performed in the full FINDERI cohort. Two binary classification tasks were considered: (1) discrimination of *APOE* ε4 non‐carriers versus carriers (0 vs ≥1 ε4 allele) and (2) discrimination of homozygous *APOE* ε4 carriers versus all other individuals (≤1 vs 2 ε4 alleles), with the latter additionally examined in a carrier‐restricted analysis as an analytical thought experiment.

ROC curves were constructed using the apoE4/pan apoE ratio as the predictor, and optimal cut points were determined using the Youden index. These ROC‐derived cut points represent in‐sample reference thresholds and were used for subsequent comparison with the manufacturer recommended cutoff.

To evaluate the performance of the upper apoE4/pan‐apoE cut point, internal validation was performed using bootstrap resampling (1000 iterations) under the two classification scenarios: discrimination of *APOE* ε4 homozygous individuals versus all others in the full cohort, and discrimination of heterozygous versus homozygous carriers within the carrier subgroup as a thought experiment. In each bootstrap iteration, classification performance was evaluated for the fixed manufacturer‐recommended cutoff (75%) and the fixed ROC‐derived cutoff (91.5%). The ROC‐derived cut points were intentionally not re‐optimized within bootstrap samples, as they were not intended for clinical implementation but served as data‐driven reference values to contextualize the performance of the manufacturer‐recommended threshold.

## 3 RESULTS

### 3.1 Study participants and apoE4 and pan‐apoE measurements in EDTA blood plasma

The study sample included 479 participants in total. The measured plasma concentrations of apoE4‐ and pan‐apoE protein and the apoE4/pan‐apoE ratio in the entire sample stratified by *APOE* genotype are summarized in Table 1. Kruskal–Wallis tests indicated statistically significant group differences in all three tested analytes.

**TABLE 1:** Characteristics of the FINDERI sample stratified by *APOE* genotype.

Characteristic	Overall *N* = 479^a^	Non‐carrier *N* = 344^a^	Heterozygous ε4 *N* = 123^a^	Homozygous ε4 *N* = 12^a^	*p*‐value^b^
***APOE* genotype**					
ε3/ε3	280 (58.5%)	280 (81.4%)			
ε2/ ε2	4 (0.84%)	4 (1.16%)			
ε2/ε3	60 (12.5%)	60 (17.4%)			
ε2/ε4	8 (1.67%)		8 (6.50%)		
ε3/ε4	115 (24.0%)		115 (93.5%)		
ε4/ε4	12 (2.51%)			12 (100.0%)	
**Age, years**	68.40 ± 8.27	68.18 ± 8.48	69.14 ± 7.86	67.00 ± 5.58	0.429
**Female sex**	98 (20.5%)	67 (19.5%)	28 (22.8%)	3 (25.0%)	0.605
**BMI**					0.366
Mean ± SD	28.24 ± 4.61	28.35 ± 4.54	27.86 ± 4.81	29.20 ± 4.48	
Median (IQR)	27.75 (24.91, 31.23)	27.77 (25.31, 31.25)	27.17 (24.39, 31.10)	28.98 (25.50, 33.05)	
(missing)	7	5	2	0	
**Charlson Comorbidity Index** ^c^	4.23 ± 1.70	4.20 ± 1.72	4.37 ± 1.63	3.67 ± 1.50	0.262
**Renal insufficiency**	62 (13.0%)	44 (12.8%)	16 (13.0%)	2 (16.7%)	0.843
(missing)	1	1	0	0	
**Acute dialysis**	3 (0.63%)	2 (0.58%)	1 (0.81%)	0 (0%)	>0.999
**MoCA (0–30)**	23.84 ± 3.57	23.84 ± 3.68	23.82 ± 3.35	24.00 ± 2.83	0.925
(missing)	5	4	1	0	
**MCI (MoCA <26)**	293 (61.8%)	210 (61.8%)	76 (62.3%)	7 (58.3%)	0.961
(missing)	5	4	1	0	
**ApoE4 (µg/mL)**					<0.001
Mean ± SD	2.53 ± 7.19	0.05 ± 0.47	6.60 ± 4.12	31.97 ± 27.03	
Median (IQR)	0.00 (0.00, 2.80)	0.00 (0.00, 0.00)	6.00 (3.50, 8.20)	22.20 (16.90, 31.55)	
Min–Max	0.00–100.00	0.00–8.40	0.00–25.60	9.80–100.00	
**Pan‐apoE (µg/mL)**					<0.001
Mean ± SD	19.70 ± 12.87	21.74 ± 13.22	13.73 ± 6.10	22.43 ± 28.21	
Median (IQR)	16.40 (12.30, 24.10)	18.40 (13.80, 25.50)	13.10 (9.60, 16.20)	11.70 (7.10, 18.45)	
Min–Max	3.90–100.00	5.90–100.00	3.90–35.50	4.30–86.80	
**ApoE4/pan‐apoE ratio**					<0.001
Mean ± SD	0.17 ± 0.38	0.00 ± 0.02	0.47 ± 0.17	1.97 ± 0.61	
Median (IQR)	0.00 (0.00, 0.30)	0.00 (0.00, 0.00)	0.46 (0.36, 0.58)	1.94 (1.71, 2.24)	
Min–Max	0.00‐3.26	0.00‐0.39	0.00‐1.02	0.95‐3.26	
**ApoE4/pan‐apoE ratio (%)**					<0.001
Mean ± SD	17.25 ± 37.58	0.19 ± 2.49	47.46 ± 16.61	196.75 ± 60.62	
Median (IQR)	0.00 (0.00, 30.00)	0.00 (0.00, 0.00)	46.00 (36.00, 58.00)	194.00 (171.00, 223.50)	
Min–Max	0.00–326.00	0.00–39.00	0.00–102.00	95.00–326.00	

^a^ Mean ± SD; *n* (%).

^b^ Kruskal–Wallis rank‐sum test; Fisher's exact test.

^c^ Charlson Comorbidity Index was calculated based on the medical history, considering factors such as myocardial infarction, heart failure, aortic aneurysm, history of stroke, and others (see Table S3).

Abbreviations: *APOE*, apolipoprotein E; BMI, body mass index; FINDERI, IQR, interquartile range; MoCA, Montreal Cognitive Assessment; MCI, mild cognitive impairment; SD, standard deviation.

Dunn's multiple comparison tests revealed that for all three tested parameters the observed group differences between (1) non‐carriers and heterozygous subjects and (2) non‐carriers and homozygous individuals reached statistical significance, but not between heterozygous and homozygous study participants (Table S1).

### 3.2 Comparison of apoE4 proteotype classification with *APOE* ε4 genotyping

**FIGURE 1:**
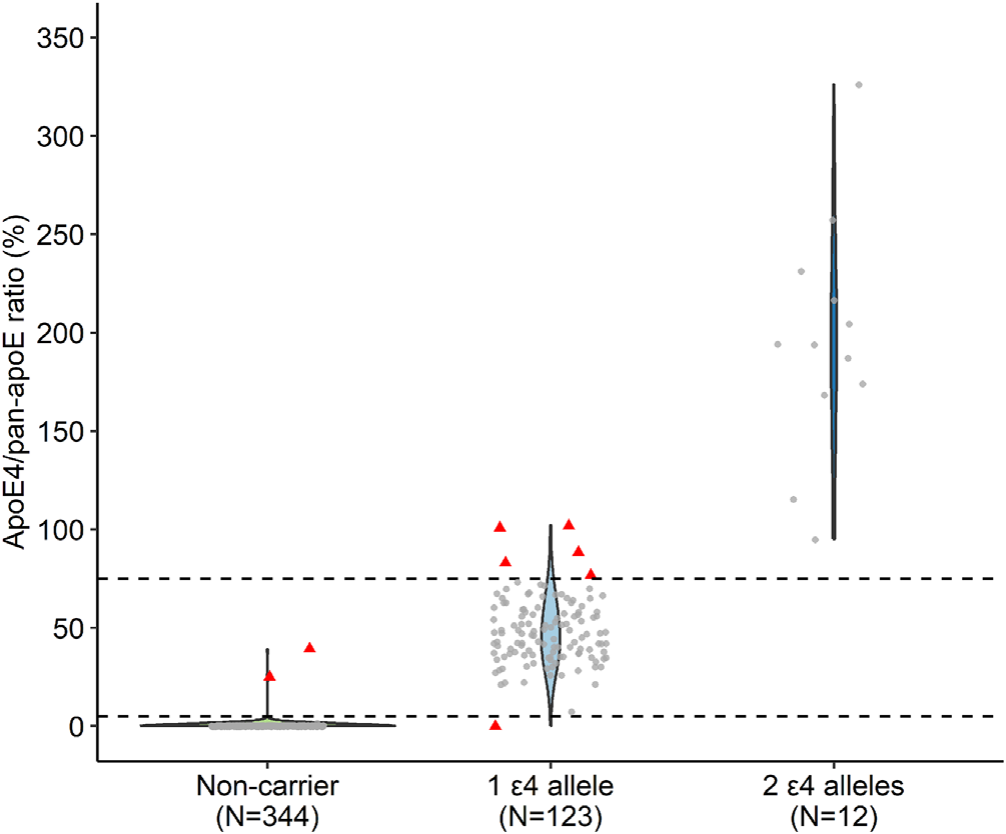
Violin plot showing plasma apoE4/pan‐apoE ratios stratified by *APOE* ε4 genotype. Dashed horizontal lines indicate the classification thresholds (non‐carrier, heterozygous, homozygous) for the apoE4/pan‐apoE ratio, as defined by the Lumipulse immunoassay. Red triangles highlight the misclassifications (*n* = 8) based on these thresholds.

Using the cutoffs provided with the assay kits, the study participants were proteotypically classified into non‐carriers (apoE4/pan‐apoE ratio <5%), heterozygous (5% ≤ ratio < 75%), or homozygous individuals (ratio ≥75%). The distribution of the calculated apoE4/pan‐apoE plasma ratios in the study sample stratified according to the *APOE* ε4 status is shown in Figure 1. We observed some overlap among the three groups, and in eight cases in total (1.67%), the apoE4 proteotype classification was discordant with the *APOE* ε4 genotype determined by qPCR analysis: Five study participants were classified as proteotype homozygous but genotyped as heterozygous, two individuals were classified as proteotype heterozygous but genotyped as non‐carriers, and one subject was classified as non‐carrier but genotyped as heterozygous. Notably, all of the genotypically homozygous cases were correctly classified by proteotyping. In cases with divergent apoE geno‐ and proteotype, genotyping was repeated, which resulted in an entire confirmation of the originally determined genotype in all investigated samples.

In the next step, the performance of the plasma apoE4/pan‐apoE ratio for the binary classification of genotypic *APOE* ε4 positivity (i.e., the presence of at least one *APOE* ε4 allele) was assessed using the binary cutoff from the Lumipulse kit instructions. Applying this classification resulted in three misclassifications (one false negative, two false positive) (Table 2). The diagnostic performance showed 99.3% sensitivity (95% confidence interval [CI] 95.94–99.98), 99.4% specificity (95% CI 97.92–99.87), and 99.4% diagnostic accuracy (95% CI 98.18–99.87) for determining *APOE* ε4 positivity. The PPV was 98.5% (95% CI 94.79–99.82) and the NPV was 99.7% (95% CI 98.39–99.99) (Table S2).

**TABLE 2:** Classification table for *APOE* ε4 presence of at least one *APOE* ε4 allele according to qPCR genotyping by the plasma apoE4/pan‐apoE ratio using the binary cutoff ≥5% apoE4/pan‐apoE ratio for apoE4 positivity.

Predicted by binary cutoff of 5%			
	*APOE* ε4 negative	*APOE* ε4 positive	Total
**Genotyping: Presence of at least one *APOE* ε4 allele**			
No	342	2	344
Yes	1	134	135
**Total**	343	136	479

To assess the performance of the plasma apoE4/pan‐apoE ratio cutoff of 75% for the classification of homozygous versus heterozygous *APOE* ε4 allele carriers, we analyzed only study participants with at least one *APOE* ε4 allele (*n* = 135, Table 3). In this subset, the cutoff had 100% sensitivity (95% CI 73.54–100.00) and 95.9% specificity (95% CI 90.77, –98.67) for identifying *APOE* ε4 homozygosity. PPV and NPV were 70.59% (95% CI 44.04–89.69) and 100.00% (95% CI 96.92–100.00), respectively (Table S2).

**TABLE 3:** Classification table for *APOE* ε4 homozygosity versus heterozygosity using the cutoff plasma apoE4/pan‐apoE ratio ≥75% = homozygous.

Cutoff 75%			
	Heterozygous	Homozygous	Total
**Genotyping**			
Heterozygous ε4	118	5	123
Homozygous ε4	0	12	12
**Total**	118	17	135

Note: *APOE* ε4 non‐carriers were excluded from this analysis.

### 3.3 Data‑driven evaluation of the upper cutoff

The ROC‐derived lower cutoff for discrimination of non‐carriers versus carriers was 4.0%, corresponding to a sensitivity of 99.3% (95% CI 97.92–99.93) and a specificity of 99.6% (95% CI 97.92–99.93). The ROC‐derived upper cutoff for discrimination of homozygous carriers versus all other individuals was 91.5%, yielding a sensitivity of 100.00% (95% CI 73.54–100.00) and a specificity of 99.6% (95% CI 97.92–99.93). Discriminative performance was high for both contrasts, with areas under the ROC curve (AUC) of 0.995 (95% CI 0.980–0.999) and 1.000 (95% CI 0.997–1.000), respectively (Table 4).

**TABLE 4:** In‐sample ROC‐derived apoE4/pan‐apoE cut points for classification of *APOE *genotype*.

Classification task	Cutoff (%)	Sensitivity	Specificity	AUC
Non‐ε4 vs ≥1 ε4 allele	4.0	99.26 (97.92–99.93)	99.57 (97.92–99.93)	0.995 (0.980–0.999)
≤1 ε4 vs 2 ε4 alleles	91.5	100.00 (73.54–100.00)	99.57 (98.46–99.95)	1.000 (0.997–1.000)

*Cut points were determined using the Youden index for discrimination of non‑carriers versus carriers and of homozygous carriers versus all other individuals. Sensitivity and Specificity are reported with 95% exact binomial confidence intervals, whereas the area under the curve (AUC) is reported alongside logit‐transformed, permutation based 95% confidence intervals.

Table 5 summarizes the bootstrap‐validated performance of the manufacturer‐recommended and data‐driven upper apoE4/pan‐apoE cut points. In the full cohort, both cut points identified all homozygous *APOE* ε4 carriers (sensitivity  =  1.00), but the data‐driven cut point achieved higher specificity and fewer false‐positive classifications. A similar pattern was observed in the carrier‐only analysis, where the manufacturer‐recommended cut point showed reduced specificity and a higher rate of false‐positive homozygote classifications among heterozygous carriers. These findings indicate that although the apoE4/pan‐apoE ratio is well suited for *APOE* ε4 genotype discrimination, the manufacturer‐recommended upper cut point, when applied to the FINDERI cohort, is conservative and may not optimally balance sensitivity and specificity across different decision scenarios, highlighting the importance of independent validation.

**TABLE 5:** Bootstrap‑validated performance of manufacturer‑recommended and data‑driven upper apoE4/pan‑apoE cut points for classification of *APOE* ε4 homozygous carriers.

Task	Metric	Data driven cutoff	Manufacturer cutoff
2 vs ≤1 ε4 allele			
(full cohort)	Accuracy	0.996 (0.990–1.000)	0.990 (0.981–0.998)
2 vs ≤1 ε4 allele			
(full cohort)	Sensitivity	1.000 (1.000–1.000)	1.000 (1.000–1.000)
2 vs ≤1 ε4 allele			
(full cohort)	Specificity	0.996 (0.989–1.000)	0.989 (0.981–0.998)
2 vs ≤1 ε4 allele			
(full cohort)	False positives (*n*)	2 (0–5)	5 (1–9)
2 vs ≤1 ε4 allele			
(full cohort)	False negatives (*n*)	0 (0–0)	0 (0–0)
2 vs 1 ε4 allele			
(allele carriers only)	Accuracy	0.985 (0.963–1.000)	0.963 (0.926–0.993)
2 vs 1 ε4 allele			
(allele carriers only)	Sensitivity	1.000 (1.000–1.000)	1.000 (1.000–1.000)
2 vs 1 ε4 alleles			
(allele carriers only)	Specificity	0.984 (0.960–1.000)	0.960 (0.921–0.992)
2 vs 1 ε4 alleles			
(allele carriers only)	False positives (*n*)	2 (0–5)	5 (1–10)
2 vs 1 ε4 alleles			
(allele carriers only)	False negatives (*n*)	0 (0–0)	0 (0–0)

*Note*: Values are reported as median (2.5th–97.5th percentile) across 1000 bootstrap iterations.

## 4 DISCUSSION

The present study evaluated the performance of fully automated Lumipulse plasma apoE4 and pan‐apoE proteotype immunoassays (Fujirebio) for estimating the *APOE* ε4 status in participants of the FINDERI study. The aim of the FINDERI study is the discovery of risk factors for delirium, cognitive decline, and dementia in cardiac surgery patients.^10^ The specific study cohort clearly distinguishes our investigation from previous studies and expands the observations to a new setting. The Lumipulse assays under investigation are for research use only and include cutoffs for defining the absence of apoE4 (apoE4/pan‐apoE <5%), heterozygosity (5% ≤ apoE4/pan‐apoE < 75%), and homozygosity (apoE4/pan‐apoE ≥75%).

In two previous studies, the Lumipulse assays demonstrated high performance in predicting the *APOE* ε4 genotype in suspected AD cases^9^ and in patients with memory complaints.^8^ Musso and colleagues investigated a small clinical sample including 39 *APOE* ε4–negative patients and 17 heterozygous and 5 homozygous individuals. The plasma apoE4/pan‐apoE ratio differed statistically significantly between the three groups, the AUC for classifying *APOE* ε4 non‐carriers versus carriers was 1.0, and the apoE4 proteotyping matched perfectly with genetic testing.^9^ In a different study, Dittrich et al. investigated a sample of individuals with memory complaints including 66 *APOE* ε4 non‐carriers and 70 heterozygous and 28 homozygous subjects.^8^ In 3 cases of 164 in total, the proteotyping based on the plasma apoE4/pan‐apoE ratio was discordant with genotyping. Two individuals with the genotype heterozygous were wrongly classified as homozygous and one genotypically *APOE* ε4–negative subject was misclassified as heterozygous.

In our present study, we included 479 heart surgery patients who were participants of the FINDERI study.^10^ The *APOE* genotype was determined by qPCR, and it turned out that 344 study participants were *APOE* ε4 negative, 123 were heterozygous, and 12 homozygous. The distribution of the plasma apoE4/pan‐apoE ratio among the three genotypic groups showed an overlap, and the observed difference between heterozygous and homozygous individuals did not reach statistical significance (*p* = 0.185). The proteotypic classification did not match with the genotype in eight cases in total: five individuals genotyped as *APOE* ε4 heterozygous were classified as homozygous, two *APOE* ε4 non‐carriers were classified as heterozygous, and one study participant with the genotype heterozygous was classified as a non‐carrier. However, ROC analyses indicated that the estimation of *APOE* ε4 homozygosity versus heterozygosity by the plasma apoE4/pan‐apoE ratio in our sample could be improved substantially when a data‐driven threshold was applied instead of the ≥75% cutoff provided by the manufacturer of the assay kits. Whether or not the new patient category investigated in our study might explain the difference in kit performance compared to previous studies^8^, ^9^ can only be speculated at this point. Other factors that might play a role include pre‐analytical variances, lot‐to‐lot differences in the assay reagents for the apoE4 and pan‐apoE immunoassays, and possibly also general assay performance. In a recent conference presentation Figdore et al. reported 100% (65/65) correct classifications with a Beckman Coulter proteotype assay but only 94% (61/65) correct classifications with the Lumipulse assays in the same sample.^14^

Taken together, our findings indicate that it may be necessary to define customized cut points depending on the population under investigation and possibly also the laboratory running the analyses. Furthermore, optimum cut points may vary depending on the specific clinical question that is addressed. For example, in the context of assessing eligibility for anti‐amyloid immunotherapy, the reliable identification of *APOE* ε4 homozygous individuals is particularly important. Very recently, at the 18th Clinical Trials on Alzheimer's Disease Conference, similar problems were reported regarding the U.S. Food and Drug Administration (FDA)–cleared Fujirebio plasma pTau217/Aβ42 assay when tested in different cohorts, which may be related to the cutoff not being universally applicable.^15^

Given the recent approval of monoclonal antibodies targeting Aβ variants for early symptomatic AD, appropriate use recommendations have been developed to guide the implementation of lecanemab^16^ and donanemab^17^ into real‐world clinical practice. Risk of ARIAs is strongly increased for *APOE* ε4 carriers and, for example, the ARIA‐H frequency has been reported to be as high as 53.6% in *APOE* ε4 homozygotes in the donanemab placebo‐controlled trials.^17^ This led the FDA to place a boxed warning on use of the antibody in this patient group and recommendations to test the *APOE* ε4 status prior to initiating a therapy.^18^ In their recommendations for appropriate use of donanemab, Rabinovici et al., suggested that assessment of the apoE proteotype is acceptable to indirectly determine the *APOE* genotype, if performed according to appropriate standards.^17^ However, our observation of five individuals in this study falsely classified as *APOE* ε4 homozygotes when applying the cutoff values provided by the supplier of the apoE proteotype assay is concerning. Given that the human medicines committee of the European Medicines Agency (EMA) has granted a marketing authorization to Leqembi and Kisunla solely in patients with one or no copy of *APOE* ε4,^6^, ^7^ these patients would have been classified not eligible for anti‐Aβ immunotherapy. However, these cases accounted for only ≈1% of our sample and all 12 genotypically *APOE ε4* homozygous study participants in our study were correctly classified by apoE proteotyping applying the cut point provided with the assay kit. Thus it appears that replacing *APOE* genotyping by proteotyping with this assay does not seem to increase the risk of inadvertently treating a patient with an anti‐amyloid antibody who actually carries two *APOE ε4* alleles. From a clinical perspective, this is of particular relevance in the context of assessing eligibility for anti‐amyloid immunotherapy.

An interesting case with discordant *APOE* ε4 genotyping and proteotyping results showing a lack of apoE4 protein expression despite an *APOE* ε2/ε4 genotype has been reported: long‐read sequencing of genomic DNA revealed a nonsense mutation resulting in a stop codon and potentially truncated protein formation.^19^ One might speculate whether in this particular case the apoE4 proteotype might be more informative for ARIA risk assessment than the genotype. In a more recent study, several *APOE* ε3/ε3 or ε3/ε4 carriers with loss‐of‐function variants were identified. For example, one *APOE* ε3/ε4 subject revealed some hyperphosphorylated tau but no appreciable Aβ pathology in the brain despite an age of 90. The underlying *APOE* variant created a truncated peptide resulting in an effective ε3/– genotype, supporting the idea that AD risk is driven by an ε4‐dependent gain of abnormal function.^20^, ^21^ More studies in larger clinical samples and cohorts undergoing Aβ immunotherapy are needed to clarify potential genotype–proteotype discrepancy and mechanisms of *APOE* ε4–linked ARIA susceptibility. The apoE proteins in the periphery and in the central nervous system represent two “distinct pools” (reviewed in^22^), and thus another important question to be investigated in the future might be if apoE4 and pan‐apoE protein measurements in both plasma and CSF may provide additional information regarding AD risk and possibly the occurrence of ARIAs on anti‐amyloid immunotherapy. Animal studies have shown that targeting *APOE* with intracerebroventricular injections of antisense oligonucleotides decreases brain apoE levels and ameliorates AD‐related pathological changes, albeit without altering liver or plasma apoE or peripheral cholesterol levels.^23^ ApoE polymorphism plays a role in lipid metabolism and influences plasma levels of apoE, lipids, and lipoproteins.^24^ In a poster presentation, Degrieck et al. reported potential interference of triglycerides with the Lumipulse pan‐apoE and apoE4 assays.^25^ Thus, one may speculate if the comorbidities among the participants of our study (see Table S3) may have an impact on the plasma apoE measurements and the calculated apoE ratio finally affecting the accuracy of the *APOE* genotype classification. This has not been investigated and might be addressed in future investigations. More studies in larger clinical samples and cohorts undergoing Aβ immunotherapy are needed to clarify potential mechanisms of *APOE* ε4–linked ARIA susceptibility.

Our study has limitations: the study cohort included only 12 *APOE* ε4 homozygous individuals in total, and the distribution of *APOE* ε4 non‐carriers, heterozygous, and homozygous study participants was skewed. Thus, our sample may not be representative of the population of patients in a clinical setting for whom eligibility for anti‐amyloid therapy or AD risk needs to be assessed. More studies in other and larger samples and specifically considering the impact of, for example, ethnicity, BMI, and comorbidities on the possibility of developing generalizable, universal cutoffs for apoE4 proteotyping immunoassays are needed.

In conclusion, our findings confirm previous studies indicating high specificity and sensitivity of the blood plasma apoE4/pan‐apoE protein ratio determined on the Lumipulse platform for estimating the *APOE* ε4 status. However, at the same time, our statistical in‐depth analysis raises doubts regarding the generalizability of the cut points provided by the manufacturer of the assay kits.

## AUTHOR CONTRIBUTIONS

Hermann Esselmann, Mohammed M. Hassan, Monika Sadlonova, and Niels Hansen collected data. Data collection was supervised by Monika Sadlonova, Ingo Kutschka, Hassina Baraki, Christine A.F. von Arnim, and Jens Wiltfang. Hans‐W. Klafki, Carlotta Derad, Maike Hohberg, and Oliver Wirths analyzed data and drafted and prepared the figures and tables with input from all authors. Thomas Asendorf, Christopher M. Celano, and Stephanie Heinemann revised the manuscript. All authors read and approved the final manuscript.

## CONFLICT OF INTEREST STATEMENT

C.M.C. has received stipends from Elsevier for editorial work for *General Hospital Psychiatry*. N.H. received funding from the Deutsche Forschungsgemeinschaft (DFG; 530229798), for traveling from Eisai GmbH, Eli Lilly, and Bristol Myers Squibb, as well as lectures honoraria from Eisai GmbH and Eli Lilly. J.W. has served on scientific advisory boards for Abbot, Biogen, Boehringer‐Ingelheim, Eli Lilly, F. Hoffmann‐La Roche, Immunogenetics, and MSD SHARP & DOHME, he has also received honorarium for lectures sponsored by Eli Lilly, Pfizer, Janssen, MSD SHARP & DOHME, Amgen, Roche Pharma, Actelion Pharmaceuticals, Guangzhou Glorylen Medical Technology Co. (China), and Beijing Yibai Science and Technology Ltd. C.A.F.vA. received honoraria from serving on the scientific advisory board of Biogen, Roche, Novo Nordisk, Biontech, Lilly, Dr. Willmar Schwabe GmbH &Co. KG, RoX Health GmbH, Bosch Health Campus, and MindAhead UG, and has received funding for travel and speaker honoraria from Lilly, Novo Nordisk, Roche, Novartis, Medical Tribune Verlagsgesellschaft mbH, Landesvereinigung für Gesundheit und Akademie für Sozialmedizin Niedersachsen e. V., FomF GmbH | Forum für medizinische Fortbildung and Dr. Willmar Schwabe GmbH & Co. KG, and research funding from the Innovationsfond (Fund of the Federal Joint Committee, Gemeinsamer Bundesausschuss, G‐BA Grants No. VF1_2016‐201; 01NVF21010; 01VSF21019), and DFG (GRK 2824: RP9; 540861493). The other authors report no competing interests. Author disclosures are available in the Supporting Information.

## ETHICS STATEMENT

All participants provided written informed consent. The study concurs with the Declaration of Helsinki, and an ethics vote was obtained when the first patient was recruited (AZ20/11/20, amendment 21/07/21). Furthermore, the study was registered as a clinical trial with the German Clinical Trials Register (DRKS00025095). A separate ethics vote (AZ9/2/2016) was obtained for the processing, storage, and preservation of blood biomaterial samples for this study.

## Supporting information




**Supplementary Table 1**: Results from Dunn's multiple comparisons tests

**Supplementary Table 2**: Diagnostic performance using the manufacturer‐recommended cutoffs, including sensitivity, specificity, positive predictive value (PPV), and negative predictive value (NPV), with 95% exact binomial confidence intervals (CIs).

**Supplementary Table 3**: Medical history of study participants

Supporting Information

## Data Availability

The datasets analyzed during the current study are available from the corresponding author on reasonable request.
