# Supplementary material for: Evaluation of fully automated ApoE4 proteotyping for APOE ε4 genotype estimation in the FINDERI cohort
Source: Alzheimers Dement (Amst). 2026 May 18;18(2):e70362. doi: 10.1002/dad2.70362 (PMC13183586; doi:10.1002/dad2.70362)
Supplement: Supplementary file 1 — Supplementary Table 1: Results from Dunn's multiple comparisons tests [file DAD2-18-e70362-s001.docx]

**Supplementary Table 1**: Results from Dunn’s multiple comparisons tests

| **ApoE4 (µg/mL)** | Mean rank diff. | Significant? | Adjusted P Value |
| --- | --- | --- | --- |
| non-carrier vs. heterozygous | -230.5 | Yes | <0.0001 |
| non-carrier vs. homozygous | -296.9 | Yes | <0.0001 |
| heterozygous vs. homozygous | -66.44 | No | 0.2188 |
|  |  |  |  |
| **Pan-apoE (µg/mL)** |  |  |  |
| non-carrier vs. heterozygous | 110.2 | Yes | <0.0001 |
| non-carrier vs. homozygous | 103.1 | Yes | 0.0336 |
| heterozygous vs. homozygous | -7.141 | No | >0.9999 |
|  |  |  |  |
| **ApoeE4/pan-apoE ratio** |  |  |  |
| non-carrier vs. heterozygous | -230.8 | Yes | <0.0001 |
| non-carrier vs. homozygous | -300.1 | Yes | <0.0001 |
| heterozygous vs. homozygous | -69.32 | No | 0.1849 |
