# Supplementary material for: Evaluation of fully automated ApoE4 proteotyping for APOE ε4 genotype estimation in the FINDERI cohort
Source: Alzheimers Dement (Amst). 2026 May 18;18(2):e70362. doi: 10.1002/dad2.70362 (PMC13183586; doi:10.1002/dad2.70362)
Supplement: Supplementary file 2 — Supplementary Table 2: Diagnostic performance using the manufacturer‐recommended cutoffs, including sensitivity, specificity, positive predictive value (PPV), and negative predictive value (NPV), with 95% exact binomial confidence intervals (CIs). [file DAD2-18-e70362-s002.docx]

**Supplementary Table 2**: Diagnostic performance using the manufacturer-recommended cutoffs, including sensitivity, specificity, positive predictive value (PPV), and negative predictive value (NPV), with 95% exact binomial confidence intervals.

| Classification task | Sensitivity | Specificity | Diagnostic accuracy | PPV | NPV |
| --- | --- | --- | --- | --- | --- |
| *APOE* ε4 positivity vs. negativity (5%) | 99.26 (95.94, 99.98) | 99.42 (97.92, 99.93) | 99.37 (98.18, 99.87) | 98.53 (94.79, 99.82) | 99.71 (98.39, 99.99) |
| *APOE* ε4 homozygosity vs. heterozygosity in *APOE* ε4 positive subset (75%) | 100.00 (73.54, 100.00) | 95.93 (90.77, 98.67) | 96.30 (91.57, 98.79) | 70.59 (44.04, 89.69) | 100.00 (96.92, 100.00) |
