# Supplementary material for: Evaluation of fully automated ApoE4 proteotyping for APOE ε4 genotype estimation in the FINDERI cohort
Source: Alzheimers Dement (Amst). 2026 May 18;18(2):e70362. doi: 10.1002/dad2.70362 (PMC13183586; doi:10.1002/dad2.70362)
Supplement: Supplementary file 3 — Supplementary Table 3: Medical history of study participants [file DAD2-18-e70362-s004.docx]

Supplementary Table 3: Medical history of study participants

Medical history

| **Characteristic** | **Overall**  N = 479^1^ | **Non E4**  N = 344^1^ | **Heterozygous E4**  N = 123^1^ | **Homozygous E4**  N = 12^1^ | **p-value**^2^ |
| --- | --- | --- | --- | --- | --- |
| **Coronary heart disease** | 353 (74.3%) | 252 (73.7%) | 90 (74.4%) | 11 (91.7%) | 0.456 |
| (missing) | 4 | 2 | 2 | 0 |  |
| **History of myocardial infarction** | 121 (25.5%) | 85 (25.1%) | 33 (26.8%) | 3 (25.0%) | 0.926 |
| (missing) | 5 | 5 | 0 | 0 |  |
| **Heart failure** | 344 (76.8%) | 252 (78.8%) | 85 (72.0%) | 7 (70.0%) | 0.282 |
| (missing) | 31 | 24 | 5 | 2 |  |
| **Heart valve vitium** | 301 (63.1%) | 224 (65.3%) | 73 (59.8%) | 4 (33.3%) | 0.056 |
| (missing) | 2 | 1 | 1 | 0 |  |
| **Aortic valve vitium** |  |  |  |  | 0.720 |
| No | 281 (59.0%) | 199 (58.2%) | 74 (60.7%) | 8 (66.7%) |  |
| Insufficiency | 64 (13.4%) | 42 (12.3%) | 20 (16.4%) | 2 (16.7%) |  |
| Stenosis | 87 (18.3%) | 68 (19.9%) | 18 (14.8%) | 1 (8.33%) |  |
| combined | 44 (9.24%) | 33 (9.65%) | 10 (8.20%) | 1 (8.33%) |  |
| (missing) | 3 | 2 | 1 | 0 |  |
| **Mitral valve vitium** |  |  |  |  | 0.300 |
| No | 273 (57.2%) | 196 (57.1%) | 67 (54.9%) | 10 (83.3%) |  |
| Insufficiency | 194 (40.7%) | 140 (40.8%) | 52 (42.6%) | 2 (16.7%) |  |
| Stenosis | 4 (0.84%) | 4 (1.17%) | 0 (0%) | 0 (0%) |  |
| combined | 6 (1.26%) | 3 (0.87%) | 3 (2.46%) | 0 (0%) |  |
| (missing) | 2 | 1 | 1 | 0 |  |
| **Tricuspid valve vitium** |  |  |  |  | >0.999 |
| No | 382 (80.4%) | 275 (80.2%) | 97 (80.8%) | 10 (83.3%) |  |
| Insufficiency | 92 (19.4%) | 67 (19.5%) | 23 (19.2%) | 2 (16.7%) |  |
| Stenosis | 1 (0.21%) | 1 (0.29%) | 0 (0%) | 0 (0%) |  |
| (missing) | 4 | 1 | 3 | 0 |  |
| **Atrial fibrillation** | 102 (21.5%) | 69 (20.4%) | 30 (24.4%) | 3 (25.0%) | 0.583 |
| (missing) | 5 | 5 | 0 | 0 |  |
| **Peripheral arterial occlusion disease** | 57 (12.0%) | 37 (10.8%) | 19 (15.6%) | 1 (8.33%) | 0.356 |
| (missing) | 3 | 2 | 1 | 0 |  |
| **Carotid stenosis** | 66 (14.1%) | 49 (14.6%) | 17 (14.0%) | 0 (0%) | 0.507 |
| (missing) | 10 | 8 | 2 | 0 |  |
| **Aortic aneurysm** | 35 (7.34%) | 26 (7.60%) | 8 (6.50%) | 1 (8.33%) | 0.876 |
| (missing) | 2 | 2 | 0 | 0 |  |
| **Aortic dissection** | 1 (0.21%) | 1 (0.29%) | 0 (0%) | 0 (0%) | >0.999 |
| (missing) | 2 | 2 | 0 | 0 |  |
| **History of endocarditis** | 7 (1.48%) | 4 (1.18%) | 3 (2.44%) | 0 (0%) | 0.491 |
| (missing) | 6 | 6 | 0 | 0 |  |
| **History of stroke** | 52 (10.9%) | 40 (11.7%) | 12 (9.84%) | 0 (0%) | 0.555 |
| (missing) | 4 | 3 | 1 | 0 |  |
| ^1^n (%) | | | | | |
| ^2^Fisher's exact test | | | | | |
